# Amelioration of PM_2.5_-induced lung toxicity in rats by nutritional supplementation with fish oil and Vitamin E

**DOI:** 10.1186/s12931-019-1045-7

**Published:** 2019-04-16

**Authors:** Juan Li, Hang Li, Haibin Li, Weili Guo, Zhen An, Xiang Zeng, Wen Li, Huijun Li, Jie Song, Weidong Wu

**Affiliations:** International Collaborative Laboratory for Air Pollution Health Effects and Intervention, School of Public Health, Xinxiang Medical University, 601 Jinsui Street, Xinxiang, 453003 Henan Province China

**Keywords:** PM_2.5_, Fish oil, Vitamin E, Lung toxicity, Inflammation, Oxidative stress

## Abstract

**Background:**

Exposure to fine particulate matter (PM_2.5_) has been associated with respiratory morbidity and mortality. Identification of interventional measures that are efficacious against PM_2.5_-induced toxicity may provide public health benefits. This study examined the inhibitory effects of nutritional supplementation with fish oil as a source of omega-3 fatty acids and vitamin E (Vit E) on PM_2.5_-induced lung toxicity in rats.

**Method:**

Sixty four male Sprague Dawley rats were gavaged with phosphate buffered saline (PBS), corn oil (5 ml/kg), fish oil (150 mg/kg), or Vit E (75 mg/kg), respectively, once a day for 21 consecutive days prior to intratracheal instillation of PM_2.5_ (10 mg/kg) every other day for a total of 3 times. Serum and bronchoalveolar lavage fluids (BALFs) were collected 24 h after the last instillation of PM_2.5_. Levels of total proteins (TP), lactate dehydrogenase (LDH), superoxide dismutase (SOD), 8-epi-prostaglandin F2α (8-epi-PGF2α), interleukin-1β (IL-1β), C-reactive protein (CRP), IL-6, and tumor necrosis factor-ɑ (TNF-ɑ) were analyzed for markers of cell injury and inflammation. Additionally, histological alterations of lung tissues were examined by hematoxylin-eosin staining.

**Result:**

Exposure to PM_2.5_ resulted in lung toxicity, represented as increased levels of total proteins, LDH, 8-epi-PGF2α, IL-1β and TNF-α, and increased infiltration of inflammatory cells, and decreased SOD in the BALFs, and systemic inflammation, as evinced by increased levels of CRP and IL-6 in serum. Strikingly, supplementation with fish oil but not Vit E significantly ameliorated PM_2.5_-induced lung toxicity and systemic inflammation.

**Conclusion:**

PM_2.5_ exposure induces oxidative stress, lung injury and inflammation, which is ameliorated significantly by fish oil and partially by Vit E.

## Background

Outdoor air pollution has become a major global public health concern. The frequency of haze, extreme air pollution episodes that are characterized by decreased visibility less than 10 km and high concentrations of PM_2.5_ (PM with an aerodynamic diameter less than 2.5 μm) and gaseous pollutants, has increased in large areas of China, including North and Central China Plains, the Yangtze River Delta, and the Pearl River Delta [1, 2]. Ambient PM, a principal component of air pollutant, has been considered as the main contributor to such haze weather [3–5]. A recent study on the global burden of diseases indicated that ambient PM_2.5_ was the fifth-ranking mortality risk factor in 2015 [6]. In Beijing, the capital of China, a 6-year period study revealed that an increase in 10 μg/m^3^ PM_2.5_ results in an elevation of 4.60% (95% CI 3.84–4.60%) and 4.48% (95% CI 3.53–5.41%) for respiratory mortality and morbidity, respectively.

Due to its small size and large surface area, PM_2.5_absorbs diverse organic, metallic and biological components and easily deposits in alveoli, leading to adverse health effects [7]. Extensive epidemiological and toxicological studies have shown that PM_2.5_ is positively associated with the incidence of respiratory disease and the exacerbation of different respiratory disease including asthma [8], chronic obstructive pulmonary disease (COPD) [9], and lung cancer [10], as well as impairment of lung function [11]. Although the mechanisms underlying PM_2.5_-induced pulmonary disorders are not fully understood, inflammation and oxidative stress are considered to play important roles in PM_2.5_-induced cardiopulmonary toxicity [12].

Omega-3 polyunsaturated fatty acids (omega-3 PFAs) found in fish oil are widely used as a nutraceutical for the prevention and treatment of cardiovascular disease and dyslipidemia. Eicosapentaenoic acid (EPA) and docosahexaenoic acid (DHA), the effective constituents of omega-3 PFAs, inhibit inflammation by modulating the release of inflammatory cytokines, such as tumor necrosis factor-alpha (TNF-α), interleukins and lipid mediators [13, 14]. Fish oil is able to compete with arachidonic acid so as to reduce the production of prostaglandin and leukotrienes [15]. Additionally, EPA and DHA are able to induce the generation of anti-inflammatory lipids, inhibit the activation of pro-inflammatory signaling through nuclear factor κB (NFκB) [16]. Vitamin E (Vit E) is regarded as an anti-oxidant substance that can block the development of some degenerative diseases by scavenging reactive oxygen species (ROS) [17, 18]. It refers to a group of eight fat soluble compounds that include four tocopherols and four tocotrienols, of which α-tocopherol (used in this study) has the highest biological activity in the eight isoforms of Vit E and terminates free radicals reactions [19, 20].

Our previous study has shown that exposure to PM_2.5_ induces oxidative stress and inflammation [21]. Given their anti-inflammatory and antioxidant properties [22], we hypothesized that fish oil and Vit E may show protective effects against PM_2.5_ toxicity. Thus, in the present study, we used the animal model to evaluate the efficacy of supplementation with fish oil or Vit E on PM_2.5_-induced lung inflammation, which would provide useful information in the design of intervention measures against PM_2.5_-induced lung toxicity.

## Materials and methods

### Reagents

Fish oil was purchased from Swiss company (Melbourne, Australia), corn oil from Shandong Sanxing corn technology Co., Ltd. (Shandong, China), and Vit E (α-tocopherol) from Sigma-Aldrich (St. Louis, USA). Fish oil, corn oil and Vit E were all certified as endotoxin free. IL-1β, IL-6, C-reactive protein (CRP), and TNF-α ELISA kits were purchased from Boster Biological Technology Co., Ltd. (Wuhan, China). 8-epi-prostaglandin F2α (8-epi-PGF2α) ELISA kit was purchased from Elabscience Biotechnology Co., Ltd. (Wuhan, China). Lactate dehydrogenase (LDH) and superoxide dismutase (SOD) assay kits were procured from Nanjing Jiancheng Bioengineering Institute (Nanjing, China).

### Animals

Sixty four 6–8 weeks old Sprague Dawley (SD) rats (specific pathogen-free grade, male, weighing 180–220 g) were purchased from Beijing Vital River Laboratory Animal Technology Co., Ltd. (Beijing, China). Rats were acclimatized for 1 week in the animal facility (19–22 °C, humidity 40–70%, 12/12-h light/dark cycle) equipped with an individual ventilated caging system prior to and during the experimental period. To avoid interference of hormonal influences with PM_2.5_ toxicity, we only used male rats in this study. All rats received food and water ad libitum. Animal use and care procedures were approved by the Institutional Animal Care and Use Committee of Xinxiang Medical University.

### PM_2.5_ collection and analyses

PM_2.5_ was collected onto quartz microfiber filters (20.3 × 25.4 cm, PALL, USA) for continuous 24 h using a PM_2.5_ high-volume air sampler (T-6070C, Tisch Environmental, USA) on the roof of the Research Building on the campus of Xinxiang Medical University (113.50° E, 35.21° N) from November 2015 to March 2016 on non-rainy days, with no large surrounding industries but heavy traffic, indicating a characteristic urban environment. The nearest main roads (Xinzhong Street and Jinsui Street) are about 120 m west and 150 m north of the sampling site, respectively. The flow rate through the impactor with a size cut at 2.5 μm was 40 cfm and continuously monitored data was recorded on a ribbon of paper. The impactor was calibrated using the flow calibrating meter (TE-5028, Tisch Environmental, USA) once a month following the manufacturer’s instructions. Before and after PM_2.5_ extraction, the quartz filters were equilibrated in a conditioning container at 22 °C and at 33% relative humid for 48 h before weighing on a microbalance (Mettler Toledo XS205, Switzerland). The PM_2.5_ was extracted from the filters through a 15 min sonication for three times with 2 min intervals. The PM_2.5_ was recovered through vacuum freeze-drying procedure and used for the intratracheal instillation. To specify the physicochemical properties of PM_2.5_, the contents of anion and metal components in PM_2.5_ were determined with ion chromatography (ICS-90, Dionex, USA) and inductive coupled plasma emission spectrometer (iCAP RQ, Thermo Fisher, USA), respectively.

### PM_2.5_ exposure and intervention

The dose of PM_2.5_ used for rat intratracheal instillation in this study was determined based on the exposure conditions of the city residents in Xinxiang: 1) The concentration of PM_2.5_ could exceed 500 μg/m^3^ in Xinxiang city on a heavy polluted day; 2) The respiratory volume is 0.13 L/min/kg for humans [23]**,** and the deposition rate of PM_2.5_ is around 40% in a human lung [24]. Based on these parameters, it is estimated that the daily (8 h) deposition of PM_2.5_ in a human lung is approximately 15.6 μg/kg body weight. Given that the conversion factor of instilled dose from humans to rats is around 10 [25], the equivalent dose for a rat is 156 μg/kg. In a preliminary study, we noticed that PM_2.5_ at 1 mg/kg only induced mild toxicity. To achieve a proper margin for evaluating the inhibitory effect of fish oil and Vit E on PM_2.5_-induced lung inflammation, we adjusted the dose of instilled PM_2.5_ in this study to 10 mg/kg referring to a similar study in which 3, 10, or 30 mg/kg of PM_2.5_ was used to assess PM_2.5_-induced cardiovascular toxicity [26]. Based on the recommended daily doses of fish oil and Vit E for an adult male and the conversion coefficient between man and rat, 150 mg/kg and 75 mg/kg body weight were used as the gavage dose of fish oil and Vit E, respectively, in this study.

Sixty four male SD rats were randomly divided into four groups according to the intervention: control group, corn oil (diluent) group, fish oil group, and Vit E group. In each group (*n* = 16), an equal number of the rats was intratracheally instilled with phosphate buffered saline (PBS) and PM_2.5_, respectively. The rats were gavaged with saline, corn oil (5 ml/kg), fish oil (150 mg/kg), and Vit E (75 mg/kg), respectively, once a day for 21 consecutive days prior to intratracheal instillation of PM_2.5_ (10 mg/kg) every other day for a total of 3 times. Normal saline was used as negative control. Corn oil was used as the diluent for fish oil and Vit E.

### Analysis of bronchoalveolar lavage fluids (BALFs)

The BALFs were collected as previously described [21]. Briefly, the rats were sacrificed 24 h after the final intratracheal instillation of PM_2.5_ suspension. The left lung was lavaged with ice-cold saline (10 ml, 5 ml per time). The recovery BALFs was approximately 80% of the amount instilled. BALFs were pooled and centrifuged at 1500 rpm for 20 min at 4 °C. The supernatants and cell pellets were collected separately. The total number of cells was counted under a light microscope.

### Histological analysis

The right lung was routinely fixed, embedded in paraffin, cut into 5 μm sections in thickness and stained with haematoxylin and eosin (H-E). The histological and morphological alterations of lung tissues were observed under a light microscope. As described above, the histology was done on a subset of non-lavaged animals through inflation fixation in accordance with the American Thoracic Society/European Respiratory Society guidelines [27].

### Examination of lung damage

LDH is a stable cytoplasmic enzyme that is present in all cells. Once plasma membrane of cell is damaged, LDH will be released instantly to the outside of the cells. Levels of LDH and total proteins in the supernatants of BALFs are proportional to the severity of lung damage and determined by LDH activity and BCA Protein Assay kit, respectively.

### Determination of inflammatory cytokines with ELISA

The blood of rats was collected and centrifuged at 684 g for 10 min at 4 °C to obtain serum. TNF-α, IL-1β, CRP and IL-6 are bio-markers of lung and systemic inflammation. Levels of TNF-α and IL-1β in BALFs as well as CRP and IL-6 in serum were determined by ELISA according to the instructions supplied by the kit’s manufacturer.

### Analysis of oxidative stress in lung

Superoxide dismutase (SOD) is a ubiquitous antioxidant enzyme that protects organisms from oxidative stress [28]. In contrast, 8-epi-PGF2α is a product of oxidative stress. Levels of SOD and 8-epi-PGF2α were used to evaluate oxidative stress using an SOD activity assay kit and 8-epi-PGF2α ELISA kit, respectively.

### Statistical analysis

ANOVA was used for multiple comparisons followed by LSD analysis. The data were presented as mean ± standard deviation (SD) and *p*-value less than 0.05 was considered statistically significant. Statistical analysis was performed using SPSS21.0.

## Results

### Water-soluble chemical composition of PM_2.5_

The concentrations of chemical constituents of PM_2.5_ are shown in Table 1. The concentrations of 11 metals (Ca, Mg, Zn, Mn, Al, Cu, Ni, Cr, Pb, Cd, Se) and seven water-soluble anions (F^−^, Ac^−^, Br^−^, Cl^−^, NO_3_^−^, HPO_4_^2−^, SO_4_^2−^, NO_2_^−^) were determined in the samples.

**Table 1:** Average mass concentration of soluble anion and metals in PM_2.5_

Metals	Mass Concentration (μg/mg PM_2.5_)	Soluble Ions	Mass Concentration (μg/mg PM_2.5_)
Ca	21.83	F^−^	4.14
Mg	3.62	Ac^−^	22.80
Zn	4.38	Cl^−^	45.56
Mn	0.20	NO_3_^3−^	0.85
Al	4.55	Br^−^	0.51
Cu	0.17	NO_3_^−^	236.41
Ni	0.09	HPO_4_^2−^	180.22
Cr	0.69	SO_4_^2−^	0.83
Pb	0.33		
Cd	0.01		
Se	0.28		

### Fish oil ameliorates PM_2.5_-induced lung damage

**Fig. 1:**
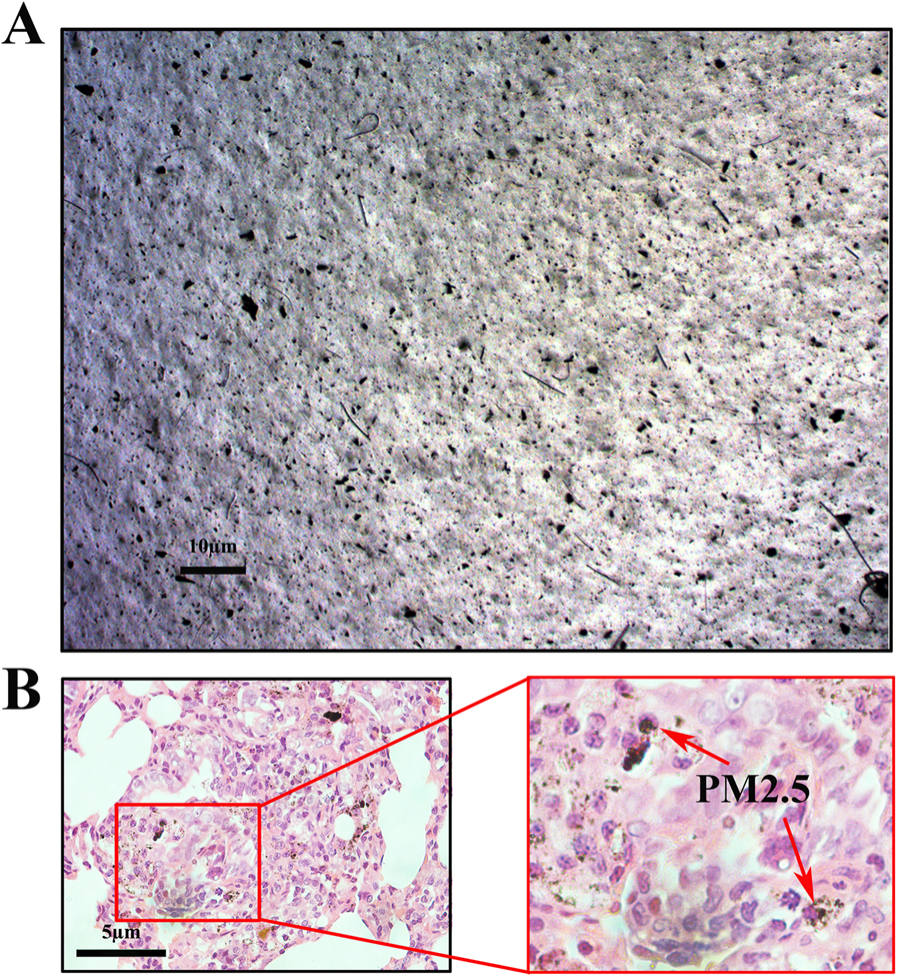
PM_2.5_ deposition in lungs of rats. **a** Fine particulate in PM_2.5_ suspension were observed using an optical microscope, bars 10 μm; (**b**) Deposition of PM_2.5_ in the deep lungs of rat, bars 5 μm. Red arrows showed that PM_2.5_ was phagocytosed by macrophage

As shown in Fig. 1a**,** a large portion of PM_2.5_ particles were seen scattered and a small portion were seen to be aggregated in a microscopic evaluation of PM_2.5_ suspension. Figure 1b showed that PM_2.5_ was mainly deposited in the alveolar area after intratracheal instillation, and that the particles were phagocytized by alveolar macrophages. Thus, these results demonstrate that PM_2.5_ is well dispersed in the suspension, which can reach alveolar areas and interact with alveolar macrophages.

**Fig. 2:**
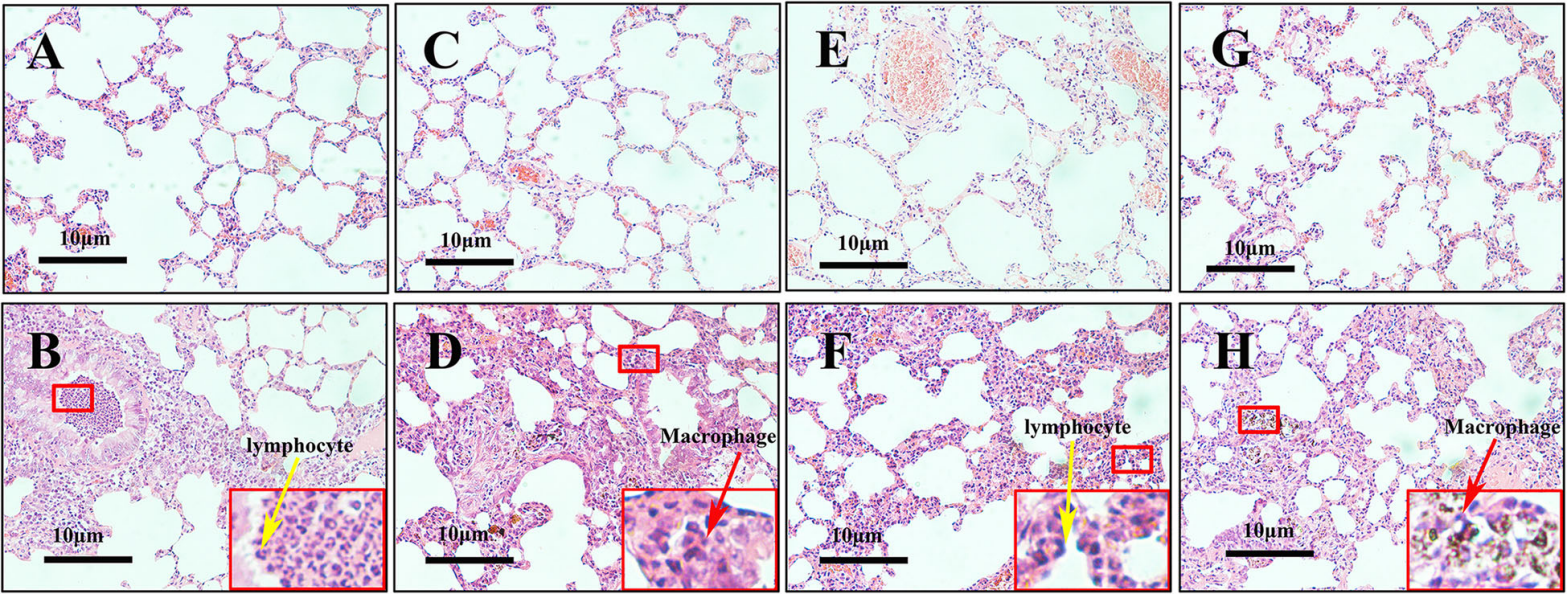
PM_2.5_-induced infiltration of inflammatory cells in lungs was ameliorated by fish oil. Pathological changes and morphological alteration of lung tissues were evaluated by HE staining. **a** PBS group; (**b**) PM_2.5_ alone group; (**c**) corn oil group; (**d**) corn oil + PM_2.5_ group; (**e**) fish oil group; (**f**) fish oil + PM_2.5_ group; (**g**) Vit E group; (**h**) Vit E + PM_2.5_ group. Yellow and red arrows showed infiltration of inflammatory cells (lymphocyte and macrophage), bars 10 μm

Fig. 2a, c, e, and g showed the architecture of lung tissues from rats only instilled with vehicle controls, including PBS, corn oil, fish oil and Vit E, respectively. Overall, these tissues showed intact lung architecture. In contrast, the lung tissues instilled with PM_2.5_ alone (Fig. 2b) induced severe inflammatory damage, represented as infiltration of inflammatory cells (macrophages and lymphocytes) into airways and surrounding tissues. Similar alterations were seen in the lung tissues instilled with PM_2.5_ in the corn oil group (Fig. 2d). In contrast, the inflammation appeared lessened in the lung tissues instilled with PM_2.5_ in the fish oil group (Fig. 2f) but not in the corn oil group (Fig. 2h). The dark matter (coalesced PM_2.5_) in the infiltrating cells in the Vit E/PM_2.5_ exposed lungs was observed (Fig. 2h). In summary, these results indicate that exposure to PM_2.5_ induces pronounced lung inflammation, which is ameliorated by supplementation with fish oil.

**Fig. 3:**
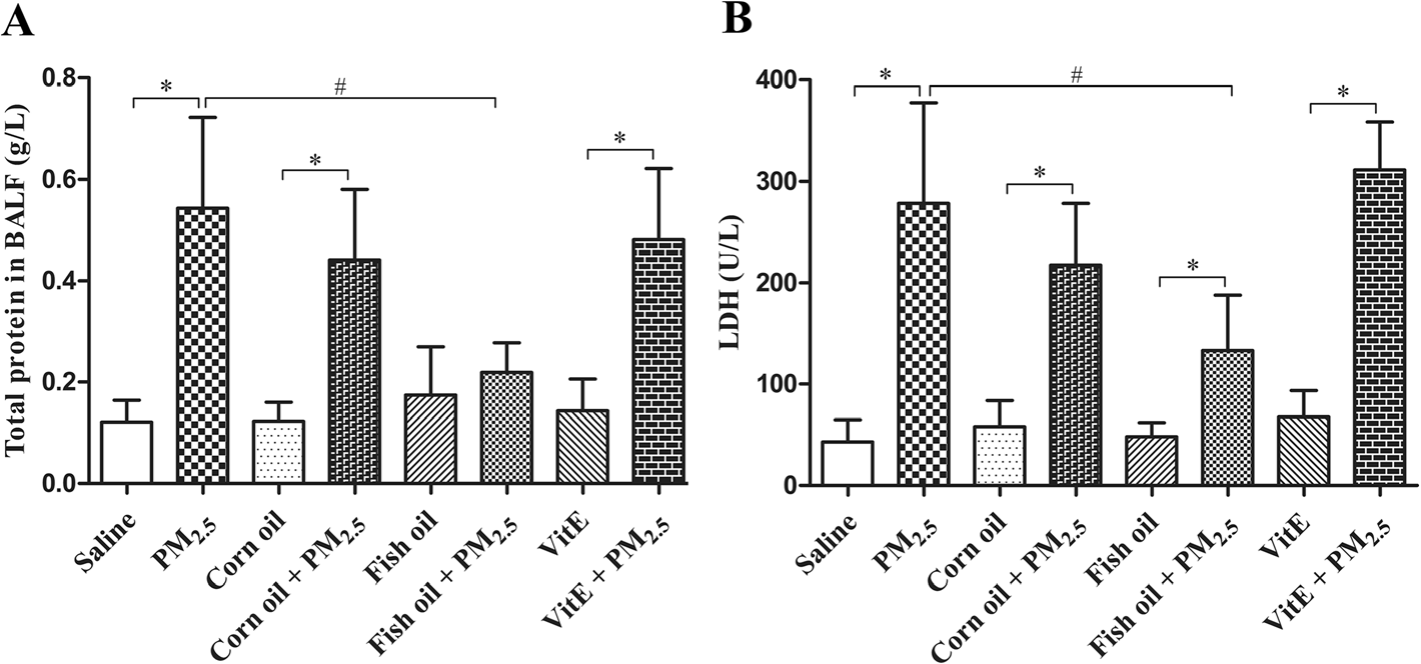
Fish oil supplementation inhibits PM_2.5_-induced TP and LDH release from rat lungs. Total protein (**a**) and LDH (**b**) were measured by using BCA protein assay kit and LDH kit, separately. All values represent the mean ± SD (*n* = 8). ^*^p < 0.05 compared with corresponding control group; ^#^*p* < 0.05 compared with PM_2.5_ group

The inflammatory alterations of rat lungs instilled with PM_2.5_ were accompanied by biochemical changes in BALF analytes, including TP and LDH, indicative of lung injury. As shown in Fig. 3, PM_2.5_ instillation increased the levels of TP and LDH in the BALFs (*p* < 0.05). This effect of PM_2.5_ was significantly blocked by supplementation of rats with fish oil (*p* < 0.05), indicating that fish oil markedly ameliorated PM_2.5_-induced lung damage. In contrast, Vit E did not show significant inhibition on PM_2.5_-induced lung damage.

### Fish oil reduces PM_2.5_-induced lung and systemic inflammatory responses

**Fig. 4:**
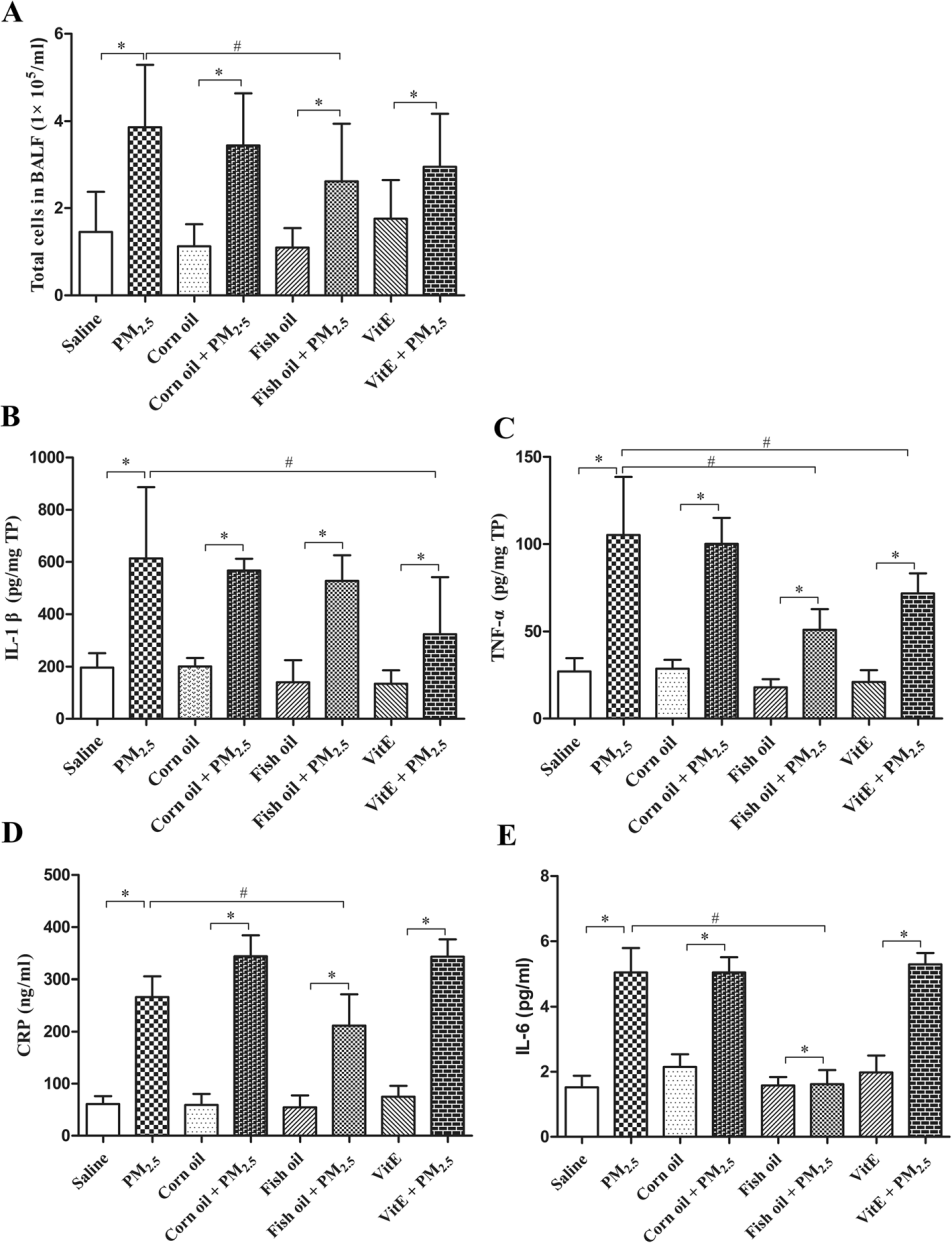
Effect of fish oil or Vit E supplementation on PM_2.5_-induced TNF-α and IL-1β release from rat lungs and CRP and IL-6 release in serum. Total inflammatory cell counts (**a**) was counted under light microscope. TNF-α (**b**), IL-1β (**c**), CRP (**d**) and IL-6 (**e**) were determined by ELISA. All values represent the mean ± SD (n = 8). ^*^p < 0.05 compared with corresponding control group; ^#^*p* < 0.05 compared with PM_2.5_ group

Total cell counts and inflammatory mediators are pivotal biomarkers of lung and systemic inflammatory responses [29, 30]. As shown in Fig. 4a, PM_2.5_ instillation significantly increased the total number of infiltrated cells, which was blocked by pre-gavage with fish oil but not with Vit E. Moreover, PM_2.5_ instillation significantly increased the levels of TNF-α and IL-1β in BALFs (*p* < 0.05), and CRP and IL-6 in serum (*p* < 0.05), as shown in Fig. 4b, c, d, and e. Supplementation with fish oil significantly blocked PM_2.5_-induced expression of TNF-α, CRP, and IL-6 (*p* < 0.05). In contrast, supplementation with Vit E only partially decreased PM_2.5_-induced IL-1β and TNF-α (Fig. 4b and c).

### Effect of fish oil and Vit E on PM_2.5_-induced oxidative stress in lungs

**Fig. 5:**
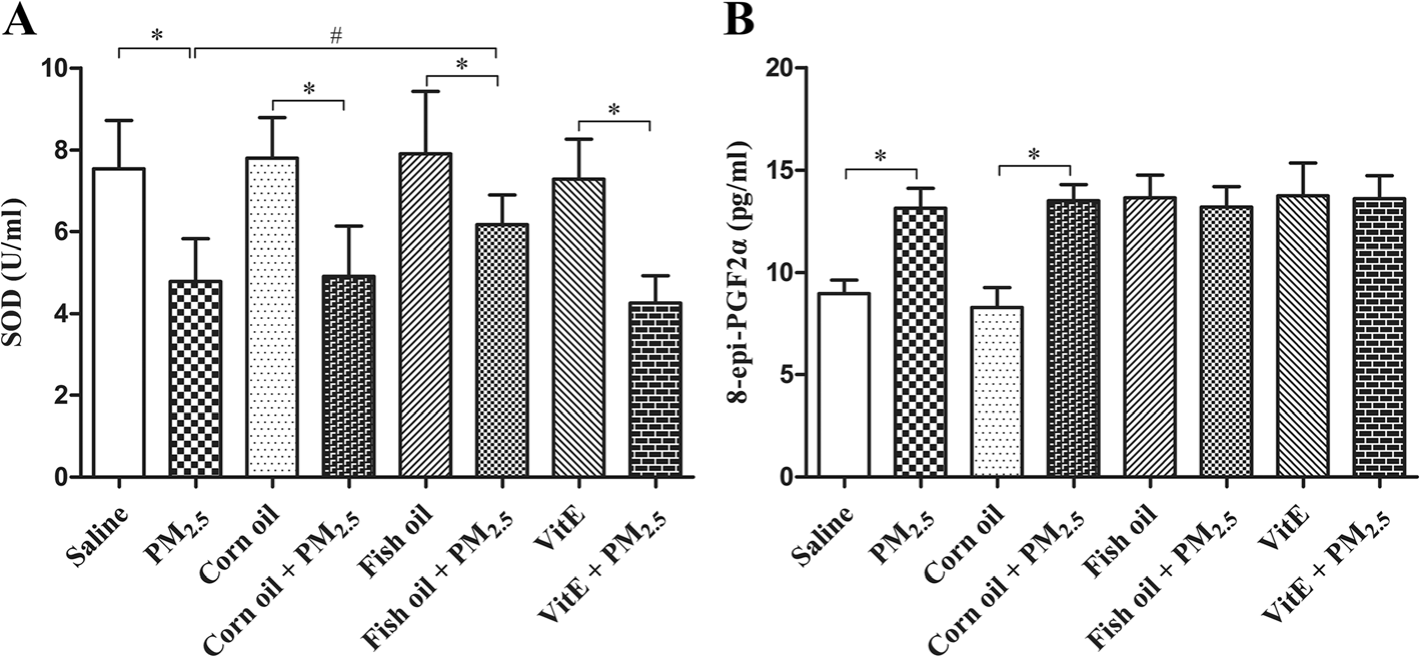
Fish oil supplementation blocks PM_2.5_-induced lung oxidative stress. SOD and 8-epi-PGF2α were measured by using superoxide dismutase detect kit and 8-epi-PGF2α ELISA kit. All values represent the mean ± SD (n = 8). ^*^p < 0.05 compared with corresponding control group; ^#^*p* < 0.05 compared with PM_2.5_ group

Oxidative stress has been proposed to be involved in PM_2.5_-induced lung toxicity [31]. Levels of SOD and 8-epi-PGF2α in BALFs were used to indirectly assess oxidative stress in rat lungs. As shown in Fig. 5a, PM_2.5_ instillation significantly decreased the levels of the antioxidant enzyme SOD, which was inhibited by supplementation with fish oil (*p* < 0.05), but not with Vit E. Instillation with PM_2.5_ markedly increased the levels of 8-epi-PGF2α in rat lung, a biomarker of oxidative stress (Fig. 5b). Interestingly, dietary supplementation with fish oil or Vit E increased the levels of 8-epi-PGF2α in both control and PM_2.5_ groups.

## Discussion

A plethora of epidemiological studies have demonstrated that exposure to PM_2.5_ is positively associated with increased prevalence of cardiovascular and respiratory disease [29, 32]. According to the global burden of disease study, 1.1 million people died from PM_2.5_ in 2015 in China [6]. In addition to the mortality costs, many areas of China have suffered from severe haze pollution recently, which greatly affects human health and daily life [1]. PM_2.5_ pollution in China, mainly from coal burning and motor vehicles emission, is linked to the rapid economic development. It is predicted that heavy PM_2.5_ pollution (haze weather) will occur frequently in some areas of China for the foreseeable future. Therefore, characterization of PM_2.5_ toxicity and potential mechanisms, and identification of effective interventional measures are of great significance to protect susceptible people against PM_2.5_ toxicity. The present study examined the inhibitory effect of fish oil and Vit E on PM_2.5_-induced lung injury and inflammation, showing that that fish oil supplementation ameliorates PM_2.5_-induced lung toxicity.

Inflammatory mediators, such as TNF-α and IL-1β, are seen to be elevated and are believed to play an important role in PM_2.5_-induced lung toxicity [5, 33, 34]. IL-1β, an important regulator of innate and acquired immune responses, is capable of recruiting inflammatory cells [35]. The function of TNF-α is associated with cell recruitment and leukocyte activation, leading to local inflammatory responses [36, 37]. Our previous study has demonstrated that PM_2.5_ induces lung inflammation, represented as increased levels of TP, IL-1β, IL-18, and cell number in the BALFs of BALB/c mice [21]. The study is further supported by the results from this study, showing that exposure to PM_2.5_ increases the levels of TP, LDH, IL-1β and total inflammatory cell number in the BALFs of rats.

In addition to lung inflammation, the results from this study also indicated that PM_2.5_ exposure induced pronounced increase in the levels of serum CRP and IL-6, indicative of systemic inflammation. However, the molecular mechanisms responsible for PM_2.5_-induced pulmonary and systemic inflammation remain unclear. Oxidative stress and inflammation are thought to play a critical role in PM_2.5_-induced lung diseases [29]. Oxidative stress is related to a biochemical imbalance process in which production of ROS exceeds the natural antioxidant capacity. ROS play an important role in PM-induced lung injury [38]. 8-epi-PGF2α has been used to indirectly assess oxidative stress [39]. SOD has been regarded as an important cellular defense for scavenging ROS [40, 41]. Therefore, the levels of SOD and 8-epi-PGF2α are regarded as a sensitive indicator of oxidative stress. In this study, exposure to PM_2.5_ decreases the levels of SOD but increased the levels of 8-epi-PGF2α in the BALFs, indicating that exposure to PM_2.5_ induces oxidative stress. The mechanisms underlying PM_2.5_-induced oxidative stress may be related to its chemical components, such as metals. In the previous study, we demonstrated that PM_2.5_ samples from different seasons rendered varied cytotoxicity (unpublished data) and the metallic components and oxidative stress seemed to play an important role in PM_2.5_-induced lung inflammatory response [42].

Inflammation and oxidative stress are critical events involved in PM_2.5_-induced adverse health effects. Given the anti-inflammatory and anti-oxidant properties of fish oil and Vit E, it is therefore envisioned that the dietary supplementation with fish oil and Vit E may act against PM_2.5_-induced lung toxicity. The recommended daily doses of fish oil and Vit E are 25 mg/kg and 14 mg/kg body weight for an adult male, respectively, according to the 2016 Chinese guideline for the management of dyslipidemia in adults. Pharmacological experiments show that the conversion coefficient between man and rat is 6.17. Therefore, 150 mg/kg and 75 mg/kg body weight were used as the gavage dose of fish oil and Vit E, respectively. The results from this study showed that fish oil supplementation significantly ameliorated PM_2.5_-induced lung damage and inflammation. Previous studies have shown that fish oil and Vit E have beneficial effect on ameliorating inflammation and oxidative stress [22]. For instance, omega-3 PFAs and Vit E decrease levels of malondialdehyde (MDA), ROS, IL-6, and TNF-α but increase SOD activity in PM_2.5_-exposed vascular endothelial cells [43]. Consistent with this study, other in vivo studies have reported that supplementation with omega-3 PFAs or Vit E inhibits PM_2.5_-induced cardiovascular injury in rats through regulation of inflammatory mediator expression and anti-oxidative activity [35, 44]. However, in this study Vit E did not significantly affect the PM_2.5_-induced lung injury (i.e., protein leak or LDH), inflammatory changes (i.e., cellular infiltration, CRP and IL-6), or oxidative stress (i.e., SOD and 8-epi-PGF2α), only partially attenuated the PM_2.5_-induced increases in IL-1β and TNF-α. These discrepancies may be partly explained by the differences in the dosage adequacy and isoforms of Vit E (i.e., α-tocopherol and γ-tocophercol), target tissues and biological parameters examined in these studies [43–46]. In addition, the way exogenous antioxidants are integrated into the physiological antioxidative defense systems may also determine their effectiveness [46].

The exact mechanisms for fish oil inhibition of PM_2.5_-induced lung inflammation have not been fully uncovered. Omega-3 PFAs as the effective component of fish oil, present immune-modulated, anti-inflammatory and anti-oxidant properties [47, 48]. They can effectively improve antioxidant metabolism and reduce the lipid peroxidation [49, 50]. Furthermore, it has been reported that omega-3 PFAs block inflammation and metabolic disorder through inhibition of pyrin domain-containing 3 (NLRP3) inflammasome activation [51]. The inflammasome, a central regulator of innate immunity and inflammation, promotes the maturation and release of several pro-inflammatory cytokines, including IL-1β, IL-18, and IL-33 [52]. ROS is regarded as the crucial elements for NLRP3 activation [53]. Exposure to PM_2.5_ has been shown to activate inflammasome and increase levels of ROS and IL-1β [5]. Inhibition of the NLRP3/Caspase1 pathway attenuates the generation of ROS and IL-1β [5]. Thus, modulation of NLRP3/Caspase1 pathway has been proposed to be another potential mechanism for fish oil inhibition of PM_2.5_-induce lung toxicity. Interestingly, dietary supplementation with fish oil or Vit E elevated the background levels of 8-epi-PGF2α in this study, implying that fish oil or Vit E increases oxidative stress. These results are consistent with previous observations that long-term intake of fish oil increases oxidative stress in women and in senescence-accelerated mice [54, 55]. The underlying mechanisms remain unclear. A previous study reported that high dose Vit E supplementary intake induced lipid peroxidation [56]. The evidence indicates the importance of rational taking nutritional supplements and their potential toxicity.

It should be mentioned that limitations exist for this study using intratracheal instillation for PM_2.5_ exposure. This approach involves a single bolus exposure of PM_2.5_ that is very different either toxicokinetically or toxicodynamically from inhalation exposure, the gold standard for evaluation of toxic effects of inhaled PM. It has been recently reported that with the same dose of nanoparticles the intratracheal instillation caused stronger and more persistent pulmonary inflammation compared with inhalation exposure [57].

## Conclusion

Given that PM_2.5_ pollution is a persistent problem in China, it is rational to search for efficacious interventional strategies to protect public health against PM_2.5_ toxicity. The present study suggests that fish oil supplementation may ameliorate PM_2.5_-induced lung toxicity, but its clinical significance needs further examination.

## Abbreviations

8-epi-PGF2α: 8-epi-prostaglandin F2α
BALF: Bronchoalveolar Lavage Fluid
COPD: chronic obstructive pulmonary disease
CRP: C-reactive protein
DHA: docosahexaenoic acid
EPA: eicosapentaenoic acid
IL-1β: interleukin-1β
LDH: Lactate Dehydrogenase
NFκB: nuclear factor κB
NLRP3: pyrin domain-containing 3
PFAs: polyunsaturated fatty acids
ROS: reactive oxygen species
SD: Sprague Dawley
SOD: superoxide dismutase
TNF-ɑ: tumor necrosis factor-ɑ
TP: total protein
VitE: Vitamin E
